# Usefulness of the second heart sound for predicting pulmonary hypertension in patients with interstitial lung disease: Utilidade da segunda bulha cardíaca na predição de hipertensão pulmonar em portadores de doenças intersticiais pulmonares

**DOI:** 10.1590/1516-3180.2015.00701207

**Published:** 2016-01-19

**Authors:** Sandra de Barros Cobra, Rayane Marques Cardoso, Marcelo Palmeira Rodrigues

**Affiliations:** I MD, MSc. Cardiologist, Hospital de Base do Distrito Federal (HBDF), Brasília, Federal District, Brazil.; II MD. Resident in General Surgery, Universidade de Brasília (UnB), Brasília, Federal District, Brazil.; III MD, MSc, PhD. Professor, School of Medicine, Universidade de Brasília (UnB), Brasília, Federal District, Brazil.

**Keywords:** Hypertension, pulmonary, Lung diseases, interstitial, Heart auscultation, Echocardiography, Phonocardiography

## Abstract

**CONTEXT AND OBJECTIVE:**

P_2_ hyperphonesis is considered to be a valuable finding in semiological diagnoses of pulmonary hypertension (PH). The aim here was to evaluate the accuracy of the pulmonary component of second heart sounds for predicting PH in patients with interstitial lung disease.

**DESIGN AND SETTING:**

Cross-sectional study at the University of Brasilia and Hospital de Base do Distrito Federal.

**METHODS:**

Heart sounds were acquired using an electronic stethoscope and were analyzed using phonocardiography. Clinical signs suggestive of PH, such as second heart sound (S_2_) in pulmonary area louder than in aortic area; P_2_ > A_2_ in pulmonary area and P_2_ present in mitral area, were compared with Doppler echocardiographic parameters suggestive of PH. Sensitivity (S), specificity (Sp) and positive (LR+) and negative (LR-) likelihood ratios were evaluated.

**RESULTS:**

There was no significant correlation between S_2_ or P_2_ amplitude and PASP (pulmonary artery systolic pressure) (P = 0.185 and 0.115; P= 0.13 and 0.34, respectively). Higher S_2_ in pulmonary area than in aortic area, compared with all the criteria suggestive of PH, showed S = 60%, Sp= 22%; LR+ = 0.7; LR- = 1.7; while P_2_> A_2_ showed S= 57%, Sp = 39%; LR+ = 0.9; LR- = 1.1; and P_2_ in mitral area showed: S= 68%, Sp = 41%; LR+ = 1.1; LR- = 0.7. All these signals together showed: S= 50%, Sp = 56%.

**CONCLUSIONS:**

The semiological signs indicative of PH presented low sensitivity and specificity levels for clinically diagnosing this comorbidity.

## RESUMO

**CONTEXTO E OBJETIVO:**

Hiperfonese de P_2_ tem sido considerada como achado valoroso no diagnóstico semiológico de hipertensão pulmonar (HP). O objetivo foi de avaliar a acurácia do componente pulmonar da segunda bulha cardíaca em predizer HP nos pacientes portadores de doenças intersticiais pulmonares.

**TIPO DE ESTUDO E LOCAL:**

Estudo transversal na Universidade de Brasília e Hospital de Base do Distrito Federal.

**MÉTODOS:**

Os sons cardíacos foram adquiridos com estetoscópio eletrônico e analisados por fonocardiografia. Os sinais clínicos sugestivos de HP, como B_2_ mais intensamente audível em área pulmonar que aórtica, P_2_ > A_2_ na área pulmonar e P_2_ presente em área mitral foram confrontados com parâmetros cardiográficos no exame de Doppler sugestivos de HP. Sensibilidade (S), especificidade (E), razões de verossimilhança positiva (RV+) e negativa (RV-) foram avaliados.

**RESULTADOS:**

Não houve correlação significativa entre amplitude de B_2_ e P_2_ e a PSAP (pressão sistólica arterial pulmonar) (P = 0,185 e 0,115; P = 0,13 e 0,34; respectivamente). A análise da presença de B_2_ mais intensa na área pulmonar que aórtica, quando comparada a todos os critérios sugestivos de HP, mostrou S = 60%; E = 22%; RV+ = 0,7; RV- = 1,7; enquanto P_2_ > A_2_ mostrou: S = 57%; E = 39%; RV+ = 0,9; RV- = 1,1; e P_2_ no foco mitral mostrou: S = 68%; E = 41%; RV+ = 1,1; RV- = 0,7. Todos os sinais juntos mostraram S = 50%; E = 56%.

**CONCLUSÃO:**

Os sinais semiológicos indicativos de HP apresentam baixos valores de especificidade e sensibilidade para diagnóstico clínico dessa comorbidade.

## INTRODUCTION

Interstitial lung diseases are a heterogeneous group of disorders that affect the lung parenchyma. However, despite their differences, they all share chronic evolution associated with functional and structural deterioration of the pulmonary parenchyma.^1^ This process is often also accompanied by pulmonary hypertension (PH), caused either by hypoxic pulmonary vasoconstriction or direct vascular impairment of vascular function, such as occurs in sarcoidosis.^2^ The presence of PH is a predictor of mortality.^3^

Detecting the presence of PH is important because this disorder is a determining factor for various therapeutic measures, among them lung transplantation.^3^ Moreover, a finding of PH may signal inapparent hypoxemia, such as occurs repeatedly during sleep or upon physical effort. Therefore, prompt and easy identification of factors that can provide additional information about the evolution of the disease is extremely important. In 1970, Harris^4^ considered that both the intensity of the second heart sound (S_2_) and its behavior during breathing deserved attention during routine auscultation. A change in its characteristics could be an early clinical sign of PH.^5^

In this scenario, splitting of S_2_ mainly occurs because of delays in the pulmonary component (P_2_), although there is a slight advance of the aortic component (A_2_). This occurs even during expiration, with a delay of 0.02 to 0.03 seconds, and 0.02 in only 2% of the population, especially in children and young adults.^6^

Hyperphonesis of P_2_ has traditionally been acknowledged in all semiology books as indicative of PH. However, there is little evidence to support this. It is defined as more accentuated presence of S_2_ in the pulmonic area than in the aortic area^5^ or, more specifically, as P_2_ > A_2_ in the pulmonic area. It shows highly variable sensitivity (S) (96% to 58%) and specificity (Sp) (46% to 19%).^5^^,^^7^

In fact, it is not uncommon for a semiological tradition to be established based on pathophysiological deductions, without proper clinical validation of the finding, including its perceived variability, which has a direct relationship with the credibility and routine application of this knowledge on a daily basis. For instance, the reliability of cardiac auscultation findings is rarely evaluated. Regardless of these issues, hyperphonesis of P_2_ is still included in the guidelines for PH as an indicator of this condition.^8^

If, on the one hand, the benefits arising from a useful clinical finding, as a means for diagnosis that is doubly accessible in terms of both cost and speed of recognition, are enormous; on the other hand, acceptance of unproven validity can be harmful to the same extent. Therefore, it is increasingly important to determine the accuracy and real reliability of these clinical findings.

## OBJECTIVE

In this study, we aimed to evaluate the pulmonary component of S_2_ as a predictor of PH in patients with interstitial lung diseases. We also attempt to determine the pulmonary artery systolic pressure (PASP) value at which the pulmonary component of S_2_ would be a more useful predictor of PH.

## METHODS

This was a cross-sectional study from March to November 2011, in which 69 patients with various interstitial lung diseases seen in the outpatient care of a tertiary-level hospital were consecutively examined. This number was defined *a priori*, assuming an effect size of 0.4 *w* for the outcome, which represents a moderate to great effect, in addition to 80% power and an α value of 5%.^9^ The study protocol was approved by our institution’s ethics committee and all the participating subjects signed an informed consent form.

Each participating patient underwent cardiac auscultation in a quiet environment, in the supine position, with spontaneous breathing. The sounds were recorded using a 3MM Littmann electronic stethoscope, model 3200 (St. Paul, MN, USA) for further analysis. Next, the patient underwent color Doppler echocardiography carried out by a single examiner who was unaware of any of the clinical data. Electrocardiographic monitoring was done during the test.

We evaluated 69 patients aged between 21 and 86 years, with a mean age of 58 ± 16.6 years. Twenty-eight subjects (40.6%) were male and 41 (59.4%), female. Regarding the distribution of diseases, 15 patients (21.7%) had idiopathic pulmonary fibrosis, 22 (32%) had idiopathic interstitial diseases, 11 (16%) had interstitial lung disease associated with collagen-vascular disease, nine (13%) had sarcoidosis, seven (10.1%) had chronic hypersensitivity pneumonia and five (7.2%) presented other diffuse interstitial lung diseases.

### Phonocardiogram

Phonocardiograms corresponding to heart sounds obtained by means of an electronic stethoscope were recorded in the aortic, pulmonic, mitral and tricuspid areas. The recording was done during spontaneous and continuous breathing.

The pulse tracings were transformed into signals by means of the Zargis Cardioscan heart sound analysis software (Princeton, NJ, USA) and were adjusted for reading in accordance with the same measurement scale. The amplitude of S_2_ was measured (with or without splitting) and the amplitude of its P_2_ component was measured separately; both measurements were obtained in the pulmonic area.

The parameters subsequently evaluated were the relative intensities of A_2_ and P_2_ in the pulmonic area; occurrences of P_2_ of greater amplitude than A_2_ (P_2_ > A_2_); P_2_ in the mitral area; absence of splitting of S_2_; and, finally, simultaneous occurrence of all the parameters.

The analyses were performed by three independent examiners. They took into consideration the sounds, the pulse tracings and the additional features of the software, which made it possible to view the spectrums of the phonographic wave forms, among other things. Decisions were then based on the consensus reached among the examiners.

Phonocardiogram results were also compared with PASP measurements by means of Doppler echocardiography, using Doppler and additional criteria for diagnosing PH, as described below.

### Transthoracic Doppler echocardiography

For transthoracic Doppler echocardiography evaluations, the patients were examined in the left lateral decubitus position, using standard echocardiographic projections. We used an ultrasound machine (model Vivid S5, General Electric. Milwaukee, WI, USA) with a multifrequency transducer and a frequency range from 2.5 to 3.5 MHz.

Measurements of variables relating to the heart chambers and ventricular function were obtained as established by the American Society of Echocardiography.^10^

Doppler analyses were performed in real time. Doppler color flow mapping in multiple views was used in order to more accurately measure tricuspid regurgitation. We used continuous wave Doppler ultrasound at a sweep speed of 50-100 mm/sec. Three to five measurements per pulse tracing were taken.

To calculate PASP by measuring tricuspid regurgitation, we used the modified Bernoulli equation. We then obtained the pressure gradient between the right ventricle (RV) and right atrium (RA). The estimated right atrial pressure was added to this parameter,^11^^,^^12^ given that there was no right ventricular outflow tract obstruction.

Right atrial pressure was obtained by assessing the percentage collapse and the diameter of the inferior vena cava during spontaneous breathing. If the inspiratory collapse was greater than 50% and the diameter was less than 2.1 cm, the pressure added was 5 mmHg; if the inspiratory collapse was less than 50% and the diameter was greater than 2.1 cm, the pressure added was 10 mmHg; in patients where the inferior vena cava plethora was markedly greater than 2.1 cm and collapse was less than 50%, the pressure added was 20 mmHg.^13^^,^^14^^,^^15^

### Pulmonary hypertension criteria

Pulmonary hypertension was considered “probable” when PASP was greater than 50 mmHg.^15^^,^^16^ It was considered “possible” when PASP fluctuated between 37 and 50 mmHg, or when it was below 37 mmHg and accompanied by additional echocardiographic variables of PH, including the existence of dilation and/or hypertrophy of the right chambers, paradoxical movement of the interventricular septum or right ventricular dysfunction (analyzed in accordance with the recommendations of the American Society of Echocardiography for evaluating the right chambers).^15^

### Data analysis

Continuous variables were described as the mean plus or minus standard deviation, along with the amplitude. Categorical variables were expressed as percentages. We conducted analyses on the correlations of the amplitudes of S_2_ and P_2_ in relation to PASP with the aim of assessing the influence of one variable on another. Since these variables did not show normal distribution according to the Shapiro-Wilk test, the Spearman correlation coefficient was used.

A receiver operating characteristic (ROC) curve with its components of sensitivity (S), specificity (Sp) and positive (LR+) and negative (LR-) likelihood ratios was constructed in order to determine the discriminatory power of each parameter studied. The area under the curve was expressed in terms of the 95% confidence interval (95% CI).

The findings were considered statistically significant when the probability P for two-tailed tests was P < 0.05. The data were analyzed using the Statistical Package for the Social Sciences (SPSS) software version 20 and Excel, both for the Mac OS X operating system.

## RESULTS

The prevalence of PH in the sample, when all the echocardiographic criteria were taken into consideration, was 73% in patients with idiopathic pulmonary fibrosis, 41% in those with idiopathic diseases, 27% in those with collagen-vascular disease, 22% in those with sarcoidosis, 25% in those with chronic hypersensitivity pneumonia and 25% in those with other diffuse lung diseases. In these patients, the forced vital capacity (FVC) showed a mean of 67 ± 22.7%, with a minimum value of 18% and maximum of 110%. Hemoglobin oxygen saturation (SpO_2_) in ambient air showed a mean of 93.4 ± 4.8% and a minimum value of 70% and maximum of 99%.

PASP estimated by means of Doppler echocardiography (which was feasible in all tests) was normal in 41 patients (59.4%), while 17 (24.7%) had additional echocardiographic criteria that, together with PASP, were suggestive of PH (possible PH). In 11 patients (15.9%), the PASP values measured by means of Doppler indicated PH (probable PH). Therefore, the combination of all the criteria measured through the examination led us to observe PH in 28 patients (40.6%).

Table 1 shows the frequencies of the clinical findings studied. For each analysis of S and Sp, we observed low values for all phonocardiographic parameters, in comparison with Doppler echocardiographic criteria, as shown in Tables 2 and 3.

**Table 1.**
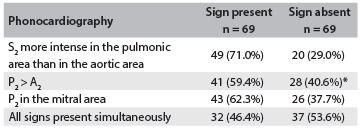
Frequencies of clinical findings surveyed

*Splitting was not observed in 9 patients (13%).

**Table 2.**
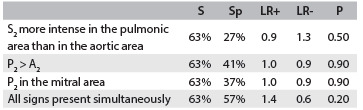
Comparison between clinical findings predictive of pulmonary hypertension (probable)

S = sensitivity; Sp = specificity; LR+ = positive likelihood ratio; and LV- = negative likelihood ratio.

**Table 3.**
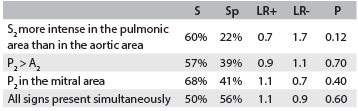
Comparison between clinical findings predictive of pulmonary hypertension (possible and probable)

S = sensitivity; Sp = specificity; LR+ = positive likelihood ratio; and LV- = negative likelihood ratio.

From observing the behavior of the maximum amplitude of S_2_ on phonocardiography (with or without splitting) and the amplitude of P_2_, in relation to the variation of PASP, we obtained a weak and not statistically significant correlation. The correlation index ρ was 0.185 for S_2_ (P = 0.13) and 0.115 for P_2_ (P = 0.34).

In assessing the ROC curve, the best cutoff point for PASP was defined as 53 mmHg. At this pressure, simultaneous presence of the three clinical signs studied showed LR+ = 2.32 and LR- = 0.88. The area under the curve was 0.518 (95% CI: 0.376 to 0.659; P = 0.80). This value was very close to the limit set for probable PH. Considering the pretest probability to be the prevalence of PH above 53 mmHg within the sample studied (which was 13%), the post-test probability would increase to 26%. For each clinical sign isolated, there were no points on the curve that yielded LR+ greater than 2 or LR- less than 0.5.

## DISCUSSION

This study included patients with several types of interstitial lung diseases, with different FVC values and degrees of hypoxemia at the time of evaluation. We observed a range of situations: normal PASP values, mild degrees of PH and also markedly elevated levels of the disease, which constituted the later stages of this comorbidity.

The prevalence of PH in these diseases varies widely according to the diagnosis and pulmonary involvement. It is also a predictor of morbidity and mortality.^17^^,^^18^^,^^19^

Doppler echocardiography has been used in other clinical studies to trace PH, in which the prevalence of this disorder was between 5.7% and 73.8% when pulmonary involvement was due to sarcoidosis.^20^ In interstitial diseases relating to collagenosis, especially scleroderma, the prevalence of PH was around 18.1%.^21^ In idiopathic pulmonary fibrosis, these data are not yet well defined, with the possibility of reaching 84% in patients with advanced degrees of pulmonary involvement.^22^ Other authors have also demonstrated its occurrence in one third of patients with IPF (interstitial pulmonary fibrosis).^23^ In the present study, the prevalence rates of PH were in agreement with the range of values previously reported.

Since the recognition of inspiratory splitting of the second heart sound by Potain^24^ 100 years ago, numerous studies have tried to explain how these heart sound variations occur and whether these changes can be attributed to various disorders. Analyses have been conducted with the aim of comparing traditional phonocardiograms with intracardiac pressure measurements made through cardiac catheterization, in order to relate pressure values to semiological findings.

There are no studies comparing intracardiac pressure measurements obtained using Doppler echocardiography with digital phonocardiogram pulse tracings obtained using an electronic stethoscope, in which patients with interstitial lung disease were specifically targeted. However, the reasons that would lead to increased PASP and possible semiological changes would be similar to those found in other diseases.

The relative intensities of heart sounds are still an integral part of auscultation. In cases of PH, the explanation for findings that the pulmonary component of the second heart sound presents greater intensity than that of the aortic component is believed to be associated with hemodynamic concepts and factors relating to the anatomy of the pulmonary artery.^25^ However, there is still controversy about the exact mechanism of this phenomenon.

Earlier studies^25^^,^^26^^,^^27^ indicated that the amplitude of the P_2_ component in PH may not differ significantly from that of A_2_. This would be explained by the fact that although the diastolic pressure gradient in the right ventricle is elevated in this condition, it would not exceed the gradient of the left chamber. In this regard, increased amplitude of the P_2_ component could only be expected in those few patients with PH in the later stages of the disease, in which the rate of increase of this gradient would be extremely high. Nevertheless, the analysis on this component did not show statistical significance.^25^

One anatomical factor that could also contribute towards greater amplitude of P_2_ in cases of PH would be greater surface area of the pulmonary valve and higher pulmonary artery distensibility, which would produce intense vibration of the semilunar valves, in comparison with the aortic valve. The combination of these factors was significant.^26^^,^^27^ The data from our study were consistent with the facts previously described and also showed no relationship between higher amplitude of the P_2_ component measured by means of phonocardiography and elevated PASP levels measured by Doppler echocardiography.

The PASP values estimated by means of color Doppler echocardiography showed a good correlation with invasive measurements (r = 0.92). The S and Sp values for predicting PH ranged from 79 to 100% for S and from 60 to 98% for Sp, in a study showing high prevalence of PH.^28^

Through evaluating the presence of clinical findings suggestive of PH and comparing the data with measurements of PASP by means of Doppler echocardiography, we noted that our values for S and Sp and the ratios for LR+ and LR- were of low clinical relevance, even when the pulmonary pressure levels were high. The findings from clinical studies that did not report any relationship between the relative intensities of the components of S_2_ found through phonocardiography and measurements of pulmonary pressure through catheterization^29^ are in agreement with these data. Other clinical findings such as P_2_ with higher amplitude than A_2_ and the presence of P_2_ in the mitral area were also compared in other studies in which pressure measurements were made by means of catheterization. There was no relationship between elevated measurements and the existence of these signs. In this context, the S and Sp values for high-amplitude P_2_ components were respectively 58-96% and 19-46%, thus demonstrating a wide variation.^5^^,^^7^

So far, the results from rigorous analysis on the S and Sp of semiological findings predictive of PH that were associated with the second heart sound have not been conclusive. However, our results showed that the discriminatory power of each of the clinical parameters evaluated was not very important for the diagnostic suspicion of PH “at the bedside”.

It should be noted that even the data from the NIH registry, which was a relevant prospective study, refer to the existence of an accentuated pulmonary component of the second heart sound, seen on clinical examination in more than 90% of the patients with PH, irrespective of its cause.^30^ However, the NIH study aimed to investigate factors associated with survival in this population. The only concern was to report the clinical findings, without determining the S and Sp of these semiological findings.

Thus, considering a context in which the prevalence of PH is high, findings of physical signs with high Sp would increase the likelihood of the disease post-test. Absence of signs showing high S would practically dismiss this possibility, and this would be useful for tracing. Our data demonstrated that these signs do not have the capacity to confirm the presence or absence of the disease. Other methods such as Doppler echocardiography are required in order to diagnose this complication.

## CONCLUSIONS

Therefore, we can conclude that, in the context of symptomatic evaluation for predicting PH in patients with interstitial diseases, clinical signs are not useful. Their pathophysiological concepts would only be useful for academic thinking. These signs cannot take on the function of reaching a diagnosis.
